# Comparative study of the 7^th^ and 8^th^ AJCC editions for gastric cancer patients after curative surgery

**DOI:** 10.1371/journal.pone.0187626

**Published:** 2017-11-13

**Authors:** Wen-Liang Fang, Kuo-Hung Huang, Ming-Huang Chen, Chien-An Liu, Yi-Ping Hung, Yee Chao, Ling-Chen Tai, Su-Shun Lo, Anna Fen-Yau Li, Chew-Wun Wu, Yi-Ming Shyr

**Affiliations:** 1 Division of General Surgery, Department of Surgery, Taipei Veterans General Hospital, Taipei, Taiwan; 2 Schoole of Medicine, National Yang-Ming University, Taipei, Taiwan; 3 Institute of Clinical Medicine, School of Medicine, National Yang-Ming University, Taipei, Taiwan; 4 Division of Medical Oncology, Department of Oncology, Taipei Veterans General Hospital, Taipei, Taiwan; 5 Department of Radiology, Taipei Veterans General Hospital, Taipei, Taiwan; 6 National Yang-Ming University Hospital, Yilan, Taiwan; 7 Department of Pathology, Taipei Veterans General Hospital, Taipei, Taiwan; National Cancer Center, JAPAN

## Abstract

**Objectives:**

The classification of pathological tumor-node-metastasis (pTNM) staging of gastric cancer was revised in the 8^th^ American Joint Committee on Cancer (AJCC) edition. The major revision was the separation of pN3a and pN3b in the pTNM staging. The current study evaluated the prognostic impact of this change.

**Methods:**

A total of 1,517 patients who underwent curative surgery for gastric cancer with a retrieved lymph node number ≥15 at our institution from January 1995 to December 2011 were enrolled. Survival was compared for the disease classified according to both the 7^th^ and 8^th^ editions.

**Results:**

After separation of pN3a and pN3b in the pTNM stage definition, the 8^th^ edition still provides significant survival differences between each stage. The multivariate analysis demonstrated that the pTNM stage in both the 7^th^ and 8^th^ editions was an independent prognostic factors of overall survival and disease-free survival. The 8^th^ edition has a better homogeneity than the 7^th^ edition with a significantly higher likelihood ratio chi-square test. Regarding the OS and DFS, the time-dependent receiver operating characteristic (ROC) curves of the two staging systems are almost overlapping, indicating that the prognostic performance is comparable between the two staging systems.

**Conclusions:**

Both the 7^th^ and 8^th^ edition-based stages are independent prognostic factors for gastric cancer. The 8^th^ edition has a better homogeneity than the 7^th^ edition; the 8^th^ edition provides discriminant survival differences among each pTNM stage that are comparable to those in the 7^th^ edition.

## Introduction

Significant survival differences between pN3a and pN3b gastric cancer have been reported [1,2]. However, pN3a and pN3b were combined into the same pathological tumor-node-metastasis (pTNM) stages in the 7^th^ American Joint Committee on Cancer (AJCC) edition. Consequently, pN3a and pN3b were separated into different pTNM stages in the new 8^th^ edition. Furthermore, pT4aN2, which was classified as stage IIIB in the 7^th^ edition, is now classified as stage IIIA in the 8^th^ edition. Moreover, pT4bN0, which was classified as stage IIIB in the 7^th^ edition, is now classified as stage IIIA in the 8^th^ edition. Finally, pT4bN2, which was classified as stage IIIC in the 7^th^ edition, is now classified as stage IIIB in the 8^th^ edition.

The aim of this study was to compare survival differences among gastric cancer patients after curative surgery according to their 7^th^ and 8^th^ AJCC edition-based stages and to investigate whether the revisions in the 8^th^ edition provide discriminative survival differences between each pNM stage comparable to those in the 7^th^ edition.

## Materials and methods

A total of 2,275 patients underwent surgery for treatment of adenocarcinoma of the stomach between January 1995 and December 2011 at our institution. The corresponding data were recorded prospectively in a gastric cancer database. Among these patients, Siewert type 2 tumors were diagnosed in 66 patients, whereas 199 patients had Siewert type 3 tumors. Sixty-one patients with Siewert type 2 tumors with involvement of the esophagogastric junction (EGJ) were excluded from the current study because their tumors were classified as esophageal cancer in the 8^th^ AJCC edition.

**Fig 1:**
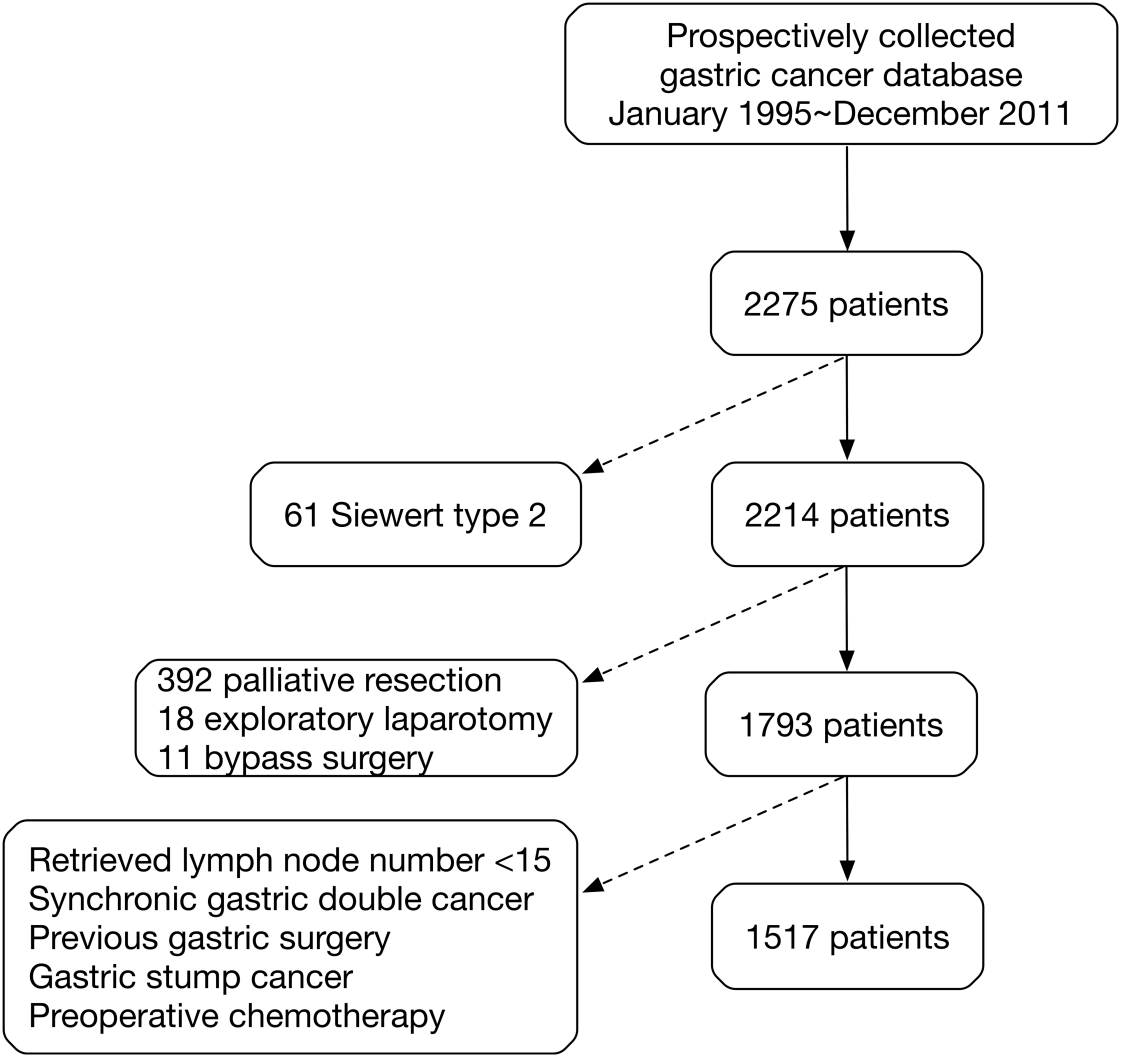
The flowchart of the enrollment of patients. The dotted arrows show the exclusion of patients.

Thus, a total of 2,214 patients were diagnosed with gastric cancer according to the 8^th^ AJCC edition. Among these patients, curative resection was performed in 1,793 patients (81.0%), palliative gastrectomy in 392 patients (17.7%), exploratory laparotomy in 18 patients (0.8%), and bypass surgery in 11 patients (0.5%). Peritoneal lavage cytology was performed for all patients who received open gastrectomy; positive cytology patients were excluded due to M1 disease and were considered to have received palliative resection. Only patients with curative resection were included. Patients with less than 15 retrieved lymph nodes were excluded to avoid inaccurate N stage induced stage migration. The exclusion criteria also included synchronous gastric double cancer, gastric stump cancer, history of gastric cancer surgery, or preoperative chemotherapy. A total of 1517 gastric cancer patients were included in this study. Among them, 5 patients had Siewert type 2 tumors and 151 patients had Siewert type 3 tumors. The enrollment of patients in this study is shown in Fig 1. The Institutional Review Board at our institution approved the present study.

Chest radiographs, an abdominal sonogram, or a computed tomography (CT) scan for tumor staging was performed for all patients. The Siewert cancer type was diagnosed by preoperative upper gastrointestinal endoscopic findings and was confirmed by operative findings.

During the operation, a total or distal subtotal gastrectomy was performed according to the tumor location. A subtotal gastrectomy was the standard procedure for distal gastric cancer, whereas a total gastrectomy was performed for proximal gastric cancer. Regarding the extent of the lymphadenectomy, a minimum of a D1+ dissection was performed for early gastric cancer, whereas at least a D2 dissection was performed for advanced gastric cancer. For the D2 dissections, a combined-organ resection was sometimes performed to achieve curative resection.

### Postoperative follow-up and management

Adjuvant chemotherapy after curative surgery was not routinely performed in our institute prior to 2008; these treatments were only applied when tumor recurrence was diagnosed or highly suspected. TS-1 adjuvant chemotherapy was started for stage II and III patients in 2008 due to its proven survival benefits [3].

Preoperative chemotherapy (n = 2) was uncommon in our institute during the study period, and was only performed for patients who were not suitable for surgery at the time of diagnosis. The two patients in this group were excluded from this study in earlier steps (one for a recent myocardial infarction and one for a recent stroke).

Follow-up visits were arranged every three months for the first five years after surgery, followed by every six months until the patient’s death. The follow-up studies included a physical examination, routine blood tests, tumor marker level assessment (e.g., carcinoembryonic antigen and carbohydrate antigen 19–9), upper gastrointestinal endoscopy, chest radiographs, an abdominal sonogram, or a CT scan. Tumor recurrence was diagnosed by biopsies or by image studies when biopsies were not obtained. Patients with tumor recurrence were eligible to receive 5-FU (Fluorouracil) based chemotherapy.

## Statistical analysis

IBM SPSS Statistics 24.0 was used for the statistical analysis. The survival analysis was performed using the Kaplan-Meier method, and a two-tailed log-rank test was used to evaluate differences between the curves. Multivariate analysis of factors affecting survival was performed using the Cox proportional hazards model. Overall survival (OS) was measured from the operation date to the date of death or the final follow-up. Disease-free survival (DFS) was defined as the length of time after gastric cancer surgery during which a patient survived without tumor recurrence. The concordance between the two systems was computed using inter-rater agreement analysis and the non-weighted kappa value. The homogeneity was analyzed by the likelihood ratio chi-square test. The multivariate logistic regression using the Cox proportional hazards model was used to evaluate the predictive capability of the competing staging systems by computing the Harrell concordance index (C-index) and Bayesian information criterion (BIC). The model with the lowest BIC was preferred. In order to quantify the improvement in risk prediction by a staging system and to evaluate its ability to discriminate patients who will develop the event of interest from those who will not, the analysis of the area-under-curve (AUC) was calculated by the time-dependent receiver-operating-characteristic (ROC) curve for censored survival data. The survival ROC analysis was performed using R statistical software. The methods of comparing the prognostic performance between different staging systems were similar to a previous study [4]. *P* values<0.05 were considered statistically significant.

## Results

### Patient characteristics

The median follow-up time was 66.7 months. The mean age of the 1,517 patients was 66.2 years (range: 23–89), and the male to female ratio was 2.28:1. A total of 801 patients (52.8%) had lymph node metastasis, and 989 patients (65.2%) had advanced gastric cancer. Among the 1,517 patients, 1,111 patients (73.2%) had at least a D2 lymph node dissection.

### Patient distributions based on the 7^th^ and 8^th^ AJCC editions

According to the 7^th^ edition, 616 patients (24.5%) were classified as stage I, including 450 staged as IA and 166 staged as IB; the same proportions were obtained according to the 8^th^ edition. Based on the 7^th^ edition, 344 (13.7%) patients were classified as stage II, with 188 staged as IIA and 156 as IIB. According to the 8^th^ edition, 341 patients (13.5%) were stage II, including 188 staged as IIA and 153 as IIB. According to the 7^th^ edition, 557 patients (22.1%) were stage III, with 150 staged as IIIA, 243 as IIIB, and 164 as IIIC. Based on the 8^th^ edition, 560 patients were stage III (22.2%), including 200 staged as IIIA, 171 as IIIB, and 189 as IIIC.

**Fig 2:**
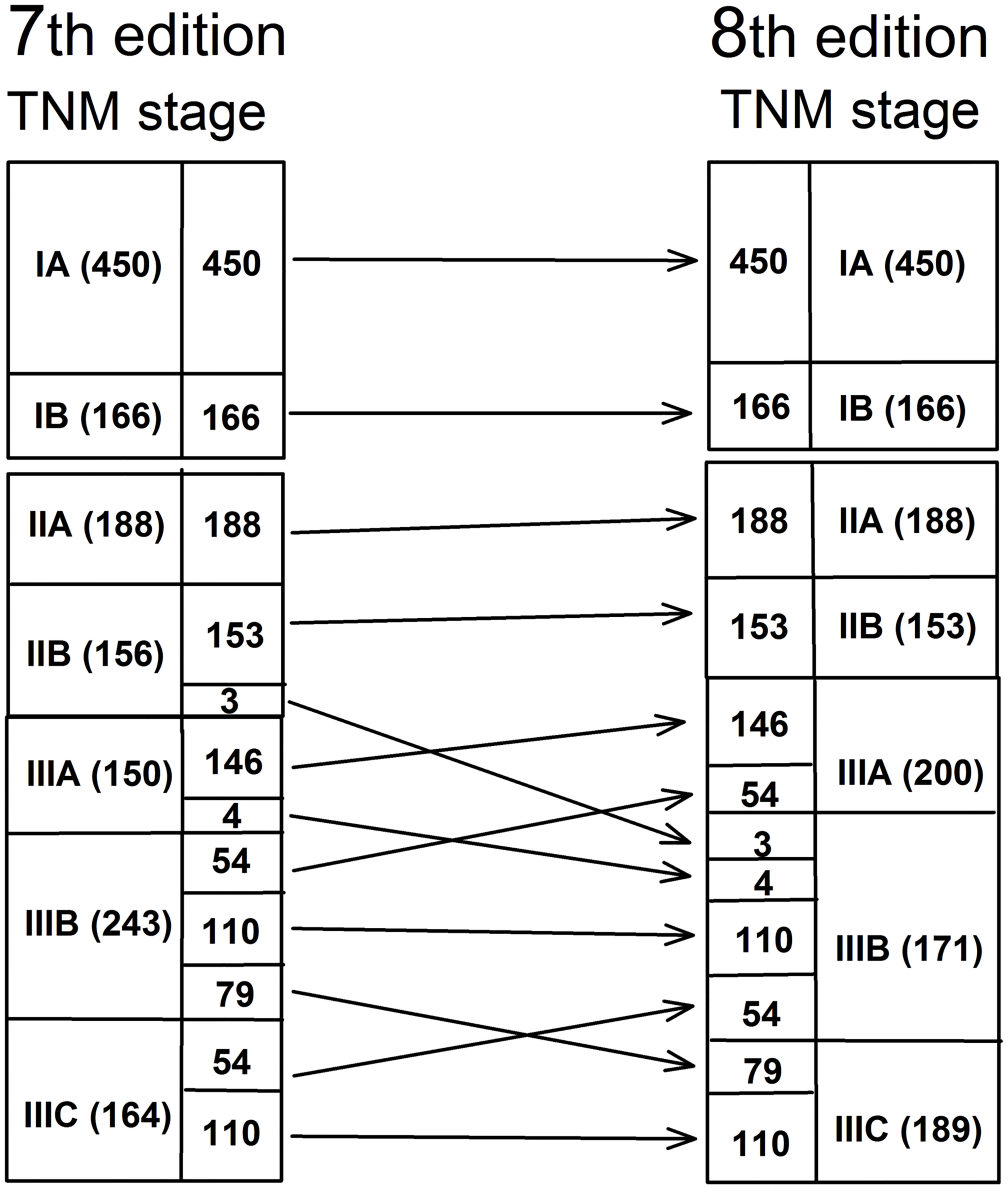
The distribution of TNM stages according to the 7^th^ and 8^th^ AJCC edition.

The changes in the tumor stages from the 7^th^ to 8^th^ edition were as follows: 3 (1.9%) stage IIB cases shifted to stage IIIB; 4 (1.3%) stage IIIA cases shifted to stage IIIB; 54 (22.2%) stage IIIB cases shifted to stage IIIA; 79 (32.5%) stage IIIB cases shifted to stage IIIC; and 54 (32.9%) stage IIIC cases shifted to stage IIIB (Fig 2).

The non-weighted kappa value was 0.820, indicating high concordance between the 7^th^ and 8^th^ editions.

### Survival rates based on the 7^th^ and 8^th^ AJCC editions

The pT and pN category definitions were the same in the 7^th^ and 8^th^ editions. The major revision in the 8^th^ edition is the separation of pN3a and pN3b in the pTNM stage.

The 5-year OS rate was 88.0% for pT1 (n = 528), 73.9% for pT2 (n = 229), 47.4% for pT3 (n = 487), and 25.6% for pT4 (n = 273) [28.2% for pT4a (n = 207) and 17.4% for pT4b (n = 66)] (*P*<0.001). The 5-year OS rate was 82.3% for pN0 (n = 716), 72.7% for pN1 (n = 210), 50.7% for pN2 (n = 226), and 21.2% for pN3 (n = 365) [27.4% for pN3a (n = 191) and 14.4% for pN3b (n = 174)] (*P*<0.001).

### Overall survival

**Fig 3:**
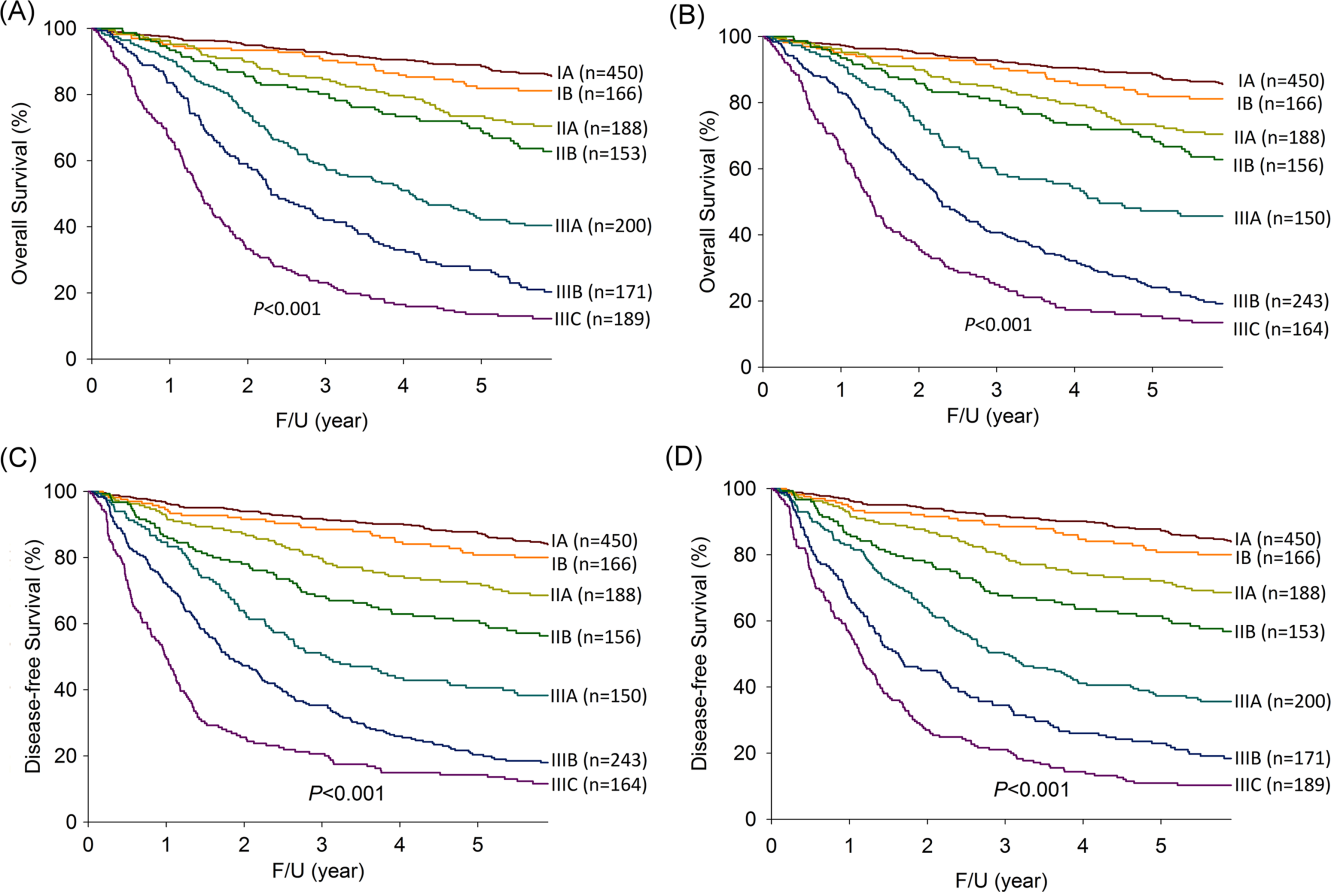
The survival curves for each gastric cancer pTNM stage. (A) Overall survival according to the 7^th^ AJCC edition; (B) Overall survival according to the 8^th^ AJCC edition; (C) Disease-free survival according to the 7^th^ AJCC edition; and (D) Disease-free survival according to the 8^th^ AJCC edition.

As shown in Fig 3A, the 5-year OS rates according to the 7^th^ edition were as follows: stage IA (88.5%); stage IB (81.9%); stage IIA (73.5%); stage IIB (68.9%); stage IIIA (47.3%); stage IIIB (24.1%); and stage IIIC (15.4%) (*P*<0.001). As shown in Fig 3B, the 5-year OS rates based on the 8^th^ edition were 88.5% for stage IA, 81.9% for stage IB, 73.5% for stage IIA, 69.0% for stage IIB, 42.1% for stage IIIA, 26.9% for stage IIIB, and 13.6% for stage IIIC (*P*<0.001).

The univariate analysis demonstrated that age, gender, tumor size, lymphovascular invasion, and the 7^th^ and 8^th^ edition pTNM stage were significantly associated with OS (Table 1). According to the 7^th^ edition, the multivariate analysis showed that age, lymphovascular invasion, and the 7^th^ edition pTNM stage were independent prognostic factors of OS (Table 2). Based on 8^th^ edition, age, lymphovascular invasion, and the 8^th^ edition pTNM stage were independent prognostic factors of OS (Table 2).

**Table 1:** A univariate analysis of factors affecting the overall survival rate.

	Univariate analysis		
	N	5-year survival rate (%)	*P* value
Age (years)			<0.001
<65	570	69.6	
≥65	947	56.9	
Gender			<0.001
Male	1055	59.2	
Female	462	67.0	
Tumor size			<0.001
< 5 cm	816	77.7	
≥ 5 cm	701	42.8	
Lymphovascular invasion			<0.001
Absent	716	81.9	
Present	801	43.5	
7^th^ AJCC edition pTNM stage			<0.001
Stage IA	450	88.5	
Stage IB	166	81.9	
Stage IIA	188	73.5	
Stage IIB	156	68.9	
Stage IIIA	150	47.3	
Stage IIIB	243	24.1	
Stage IIIC	164	15.4	
8^th^ AJCC edition pTNM stage			<0.001
Stage IA	450	88.5	
Stage IB	166	81.9	
Stage IIA	188	73.5	
Stage IIB	153	69.0	
Stage IIIA	200	42.1	
Stage IIIB	171	26.9	
Stage IIIC	189	13.6	

**Table 2:** Multivariate analysis of the prognostic factors for overall survival.

	Multivariate analysis		
	HR	95% CI	*P* value
Overall survival			
7^th^ AJCC edition			<0.001
IA	Ref		
IB	1.063	0.696–1.155	
IIA	1.470	0.890–1.466	
IIB	1.693	1.024–1.683	
IIIA	2.483	1.510–2.522	
IIIB	4.837	2.509–4.044	
IIIC	6.675	4.039–6.594	
Age	1.031	1.640–2.133	<0.001
Gender	1.114	1.040–1.383	0.217
Tumor size	1.114	1.073–1.402	0.207
Lymphovascular invasion	1.475	1.081–1.477	<0.001
Overall survival			
8^th^ AJCC edition			<0.001
IA	Ref		
IB	1.080	0.781–1.491	
IIA	1.510	1.117–2.041	
IIB	1.728	1.263–2.365	
IIIA	2.751	2.050–3.693	
IIIB	4.993	3.694–6.749	
IIIC	8.490	6.220–11.590	
Age	1.033	1.026–1.040	<0.001
Gender	1.126	0.748–1.054	0.173
Tumor Size	1.099	0.928–1.301	0.274
Lymphovascular invasion	1.422	1.167–1.733	<0.001

AJCC: American Joint Committee on Cancer; C-index: Harrell concordance index

#### Prognostic performance for OS

The likelihood ratio chi-square test was 685.26 for the 7^th^ edition and 707.53 for the 8^th^ edition (*P*<0.001), showing that the 8^th^ edition has a better homogeneity than the 7^th^ edition. Validation analyses were performed to compare the 7^th^ and 8^th^ AJCC staging systems. Regarding the OS, the C-index of the 7^th^ AJCC edition did not differ significantly from that of the 8^th^ AJCC edition (0.7345 vs. 0.7359, *P* = 0.239). The BIC was 17106.04 for the 7^th^ edition and 17083.77 for the 8^th^ edition (Table 3).

**Table 3:** Comparison of the prognostic performance using C-index, BIC, and likelihood ratio.

	7^th^ AJCC edition	8^th^ AJCC edition	P value
Overall survival			
C-index	0.7345	0.7359	0.239
BIC	17106.04	17083.77	
Likelihood ratio	685.26	707.53	<0.001
Disease-free survival			
C-index	0.7347	0.7361	0.325
BIC	17585.54	17597.16	
Likelihood ratio	728.51	740.13	<0.001

AJCC: American Joint Committee on Cancer; C-index: Harrell concordance index; BIC: Bayesian information criterion

**Fig 4:**
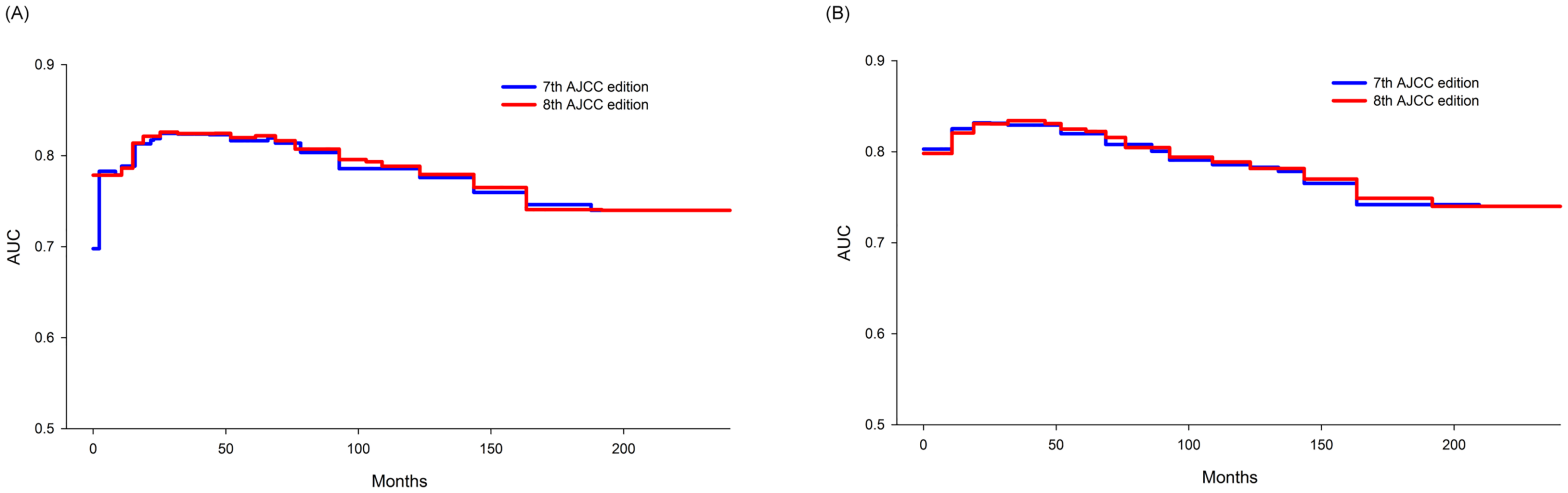
The time-dependent ROC analysis for survival data. (A) Overall survival according to the 7^th^ and 8^th^ AJCC editions; (B) Disease-free survival according to the 7^th^ and 8^th^ AJCC editions.

Comparison of the time-dependent ROC curves for the OS in 7^th^ and 8^th^ editions was shown in Fig 4A. The two ROC curves seemed almost overlapped each other, showing that the prognostic performance was similar between the two staging systems.

### Disease-free survival

As shown in Fig 3C, the 5-year DFS rates with staging according to the 7^th^ edition were as follows: stage IA (87.3%); stage IB (80.8%); stage IIA (72.1%); stage IIB (60.9%); stage IIIA (40.6%); stage IIIB (20.4%); and stage IIIC (14.3%)(*P*<0.001). As shown in Fig 3D, the 5-year DFS rates with stage according to the 8^th^ edition were as follows: stage IA (87.3%); stage IB (80.8%); stage IIA (72.1%); stage IIB (61.5%); stage IIIA (37.3%); stage IIIB (23.0%); and stage IIIC (10.9%)(*P*<0.001).

The univariate analysis showed that age, gender, tumor size, lymphovascular invasion, and the 7^th^ and 8^th^ edition pTNM stage correlated significantly with DFS. Based on the 7^th^ edition, the multivariate analysis showed that age, lymphovascular invasion, and the 7^th^ edition pTNM stage were independent prognostic factors of DFS (Table 4). According to the 8^th^ edition, the multivariate analysis demonstrated that age, lymphovascular invasion, and the 8^th^ edition pTNM stage were independent prognostic factors of DFS.

**Table 4:** Multivariate analysis of the prognostic factors for disease-free survival.

	Multivariate analysis		
	HR	95% CI	*P* value
Disease-free survival			
7^th^ AJCC edition			<0.001
IA	Ref		
IB	0.999	0.728–1.372	
IIA	1.444	1.082–1.926	
IIB	1.862	1.383–2.508	
IIIA	2.735	2.030–3.685	
IIIB	4.575	3.470–6.034	
IIIC	6.706	4.981–9.028	
Age	1.677	1.429–1.967	<0.001
Gender	1.235	1.046–1.458	0.013
Tumor size	1.135	0.965–1.334	0.126
Lymphovascular invasion	1.454	1.205–1.755	<0.001
Disease-free survival			
8^th^ AJCC edition			<0.001
IA	Ref		
IB	1.015	0.739–1.393	
IIA	1.481	1.109–1.977	
IIB	1.888	1.397–2.550	
IIIA	2.886	2.177–3.827	
IIIB	5.014	3.752–6.699	
IIIC	7.825	5.794–10.567	
Age	1.018	1.010–1.025	<0.001
Gender	0.883	0.720–1.084	0.235
Tumor size	1.124	0.955–1.321	0.159
Lymphovascular invasion	1.404	1.161–13698	<0.001

AJCC: American Joint Committee on Cancer; C-index: Harrell concordance index

#### Prognostic performance for DFS

The likelihood ratio chi-square test was 728.51 for the 7^th^ edition and 740.13 for the 8^th^ edition (P<0.001), indicating that the 7^th^ edition has a batter homogeneity than the 8^th^ edition. Validation analyses were performed to compare the 7^th^ and 8^th^ AJCC staging systems. Regarding the DFS, the 7^th^ AJCC edition had a C-index of 0.7347 compared with a C-index of 0.7361 for the 8^th^ AJCC edition (*P* = 0.325). The BIC was 17597.16 for the 7^th^ edition and 17585.54 for the 8^th^ edition (Table 3).

Comparison of the time-dependent ROC curves for the DFS in 7^th^ and 8^th^ editions was shown in Fig 4B. It seems that the two ROC curves were almost overlapping, indicating that the two systems have a similar prognostic performance.

## Discussion

The present study shows that staging according to both the 7^th^ and 8^th^ AJCC editions is an independent prognostic factor of OS and DFS. Prognostic performance studies demonstrated that both the 7^th^ and 8^th^ editions provided discriminant survival differences among each TNM stage for OS and DFS. The likelihood ratio chi-square test showed the 8^th^ edition has a better homogeneity than the 7^th^ edition. The time-dependent ROC curves of the two staging systems are almost overlapping with regard to both OS and DFS, indicating the prognostic performance is comparable between the two staging systems.

Previous studies reported that N3a and N3b should be separated in the pTNM stage in the 7^th^ edition due to significant survival differences [1,2]. Thus, this modification decision regarding the separation of pN3a and pN3b in the 8^th^ edition appears accurate. In the subgroup analysis, the patient numbers were insufficient when survival differences between pN3a and pN3b in pT1 or pT2 tumors were compared; therefore, we could only compare the survival differences between pN3a and pN3b in pT3 and pT4 tumors. In the pT3 tumors, the 5-year overall survival rate was 26.5% for the pN3a tumors (n = 106) and 17.1% for the pN3b tumors (n = 79); this difference was not significant (*P* = 0.110), although the survival curves showed a trend for better survival with pN3a tumors than with pN3b tumors (S1 Fig). More patients may be required to obtain significant survival differences between pT3N3a and pT3N3b tumors. In pT4a tumors with lymph node metastasis, the 5-year overall survival rate was 48.3% for pN1 tumors, 27.7% for pN2 tumors, 25.6% for pN3a tumors, and 10.2% for pN3b tumors (*P*<0.001, S2 Fig). The 5-year overall survival did not differ significantly between the pT4bN3a and pT4bN3b tumors (17.1% vs. 9.1%, *P* = 0.381). Thus, in the 8^th^ edition, separating pT4aN1, pT4aN2, pT4aN3a and pT4aN3b tumors into different pTNM stages was reasonable, whereas pT4bN3a and pT4bN3b remained in the same stage IIIC. Our results show that the significant survival difference is limited to only T4a. Thus, the change in N3 determination may not provide clinical relevance because we do not have different treatment methods of these gastric cancer stages at present.

Notably, pT4bN0 and pT4bN1 were classified as stage IIIB in the 7^th^ edition but the former was revised to stage IIIA in the 8^th^ edition. Additionally, pT4bN2, pT4bN3a, and pT4bN3b were classified as stage IIIC in the 7^th^ edition but were revised to stage IIIB in the 8^th^ edition. According to our database, the 5-year overall survival rates were 36.4% for pT4bN0 and 45.5% for pT4bN1, which was not a significant difference (*P* = 0.548). Additionally, the 5-year overall survival rates were 12.5% for pT4bN2, 13.3% for pT4bN3a and 6.5% for pT4bN3b, which were not significant differences. Because our results do not show significant survival differences for the revisions in the 8^th^ edition described above, more enrolled patients and analyses with other studies are still required to determine whether the revisions are meaningful and necessary. Indeed, the AJCC tumor staging definitions were made according to large data analyses. We hope our results might provide useful information for future AJCC tumor staging revisions.

In the present study, the multivariate analysis demonstrated that both the 7^th^ and 8^th^ edition-based stages were independent prognostic factors of OS and DFS. To compare the performance between the 7^th^ and 8^th^ editions, the likelihood ratio chi-square test showed a better homogeneity for the 8^th^ than the 7^th^ edition. Further validation studies including the C-index, BIC, and time-dependent ROC curve were utilized. Regarding the prognostic performance, the C-index showed no significant difference between the two staging systems; whereas, the BIC values demonstrated a slightly better prognostic performance for the 8^th^ edition compared to the 7^th^ edition regarding the OS, and a slightly better prognostic performance for the 7^th^ edition compared to the 8^th^ edition regarding the DFS. However, the most widely used method, time-dependent ROC curves, showed that the 8^th^ edition provided survival discrimination that was comparable to the 7^th^ edition. Our results may raise a question concerning whether there is a need for the recent change in the 8th edition of the AJCC staging system for gastric cancer, because this edition is so similar to the 7th edition. Should we continue using the 7th edition, since the change in N3 determination adds little to clinical management? We should be careful with the answer to this question. Although the revision might be not perfect and satisfactory, it is considered the most appropriate change at present. We believe that there will be a revision in the future regarding the N3 determination. Worldwide validation is required for the new edition, which aims to better predict survival and provide better treatment for patients.

In addition to N3 separation, another important change is the classification of EGJ tumors. Sano et al [5] reported the new stage grouping of the IGCA (The International Gastric Cancer Association) with enrollment of a large series of gastric cancer patients from Eastern and Western countries. The definitions of the T categories, N categories and TNM staging according to the IGCA stage grouping are the same as in the 8^th^ edition. However, Sano et al did not investigate whether the new staging system had a better prognostic performance than the 7^th^ edition. For EGJ tumors (n = 1117) in the large series, the authors recommended the use of the gastric cancer staging for both Siewert type 2 and 3 tumors according to the IGCA stage grouping instead of the esophageal or gastric cancer staging according to the 7^th^ edition due to better survival stratification. However, in the 8^th^ edition, Siewert type 2 tumors with EGJ (so-called Z-line) invasion are classified as esophageal cancer, whereas Siewert type 2 tumors without EGJ invasion and Siewert type 3 tumors are classified as stomach cancer, which is different from the recommendation of the IGCA group. Practically speaking, most institutions cannot easily retrospectively define whether their patients with Siewert type 2 or 3 tumors have EGJ invasion or not. In our clinical practice, we took photographs and sketches of all of the resected specimens for each patient; as a result, we can precisely classify the Siewert type and describe the relationship to EGJ. Among the 66 Siewert type 2 tumors during the study period, only 5 tumors (7.6%) had no EGJ invasion and were classified as stomach cancer, including two stage I, one stage II, and two stage III tumors. Siewert type 1 cancer is generally treated as esophageal cancer and Siewert type 3 as gastric cancer. However, due to the location of Siewert type 2 caner, there has been controversy regarding the type of surgical approach, extent of lymphadenectomy, and even use of the staging system of esophagus or stomach. In Western countries, Siewert type 1 tumors have increased and become more predominant over the past decades, whereas in Eastern countries, Siewert type 2 and 3 cancers are far more common than Siewert type 1 cancer. In our previous study regarding EGJ tumors [6], the overall survival of patients with Siewert type 2 tumors was not significantly different from the overall survival of patients with Siewert type 3 tumors, which might indicate similar tumor behavior between Siewert type 2 and 3 tumors. According to the recommendation of the IGCA group [5] and due to the difficulty of retrospectively clarifying the location of Siewert type 2 or 3 tumors in relation to EGJ in most institutions, both Siewert type 2 and 3 tumors may be better classified as gastric cancer regardless of EGJ invasion.

Although adjuvant chemotherapy with TS-1 was recommended for stage II and III patients [3], fewer than 50% of our patients received TS-1 therapy between 2008 and 2011 because the patients needed to pay for these treatments on their own. Since 2017, TS-1 has been approved as an adjuvant chemotherapy after curative gastric cancer surgery by health insurance in our country; as a result, the percentage of adjuvant chemotherapy with TS-1 has increased dramatically. Regarding EGJ tumors in our institute between 2008 and 2011, three Siewert type 2 tumors were diagnosed as stage II or III, for which one patient (33%) received adjuvant chemotherapy, and twenty-two Siewert type 3 tumors were diagnosed as stage II or III, for which eleven patients (50%) received adjuvant chemotherapy.

Based on our observations, patients who received D3 dissection (para-aortic lymph node dissection) prior to 2006 had a higher surgery-related complication rate than patients who received D2 dissection (26.8% vs. 16.3%, *P* = 0.001), which was similar to other reported results [7,8]. D3 dissection most likely had no survival benefit compared to D2 dissection for our patients (46.8% vs. 53.5%, *P* = 0.259), which was confirmed by the results of other studies [9,10]. In 2006, we stopped performing D3 dissection, and D2 dissection became the routine procedure for advanced gastric cancer in our service.

The present study has several limitations. First, this study was a single-institution study, and a selection bias may exist. Second, the patient number was not sufficiently large to perform a subgroup analysis. For example, only three patients had pT1N3b tumors and four patients had pT2N3b tumors in the present study. Future studies with more patients enrolled for the subgroup analysis are required.

In conclusion, the 8^th^ edition has a better homogeneity than the 7^th^ edition, and the 8^th^ AJCC edition is comparable to the 7^th^ edition with respect to the discrimination of survival differences among each pTNM stage. However, the revisions regarding pT1N3b, pT2N3b, pT3N3b, pT4bN0, and pT4bN2 in the pTNM staging in the 8^th^ edition require more patients and more studies for validation. Furthermore, we recommend classifying both Siewert type 2 and 3 tumors as gastric cancer regardless of EGJ invasion. We anticipate that our results will provide a reference for future revisions of the AJCC staging system.

## Supporting information

S1 Fig The 5-year overall survival rates for pT3N3a and pT3N3b gastric cancer. (DOCX)

S2 Fig The 5-year overall survival rates for pT4aN1, pT4aN2, pT4aN3a, and pT4aN3b gastric cancer. (DOCX)
