# Supplementary figures and images for: Comparative study of the 7th and 8th AJCC editions for gastric cancer patients after curative surgery
Source: PLoS One. 2017 Nov 13;12(11):e0187626. doi: 10.1371/journal.pone.0187626 (PMC5683565; doi:10.1371/journal.pone.0187626)

**S1 Fig.** The 5-year overall survival rates for pT3N3a and pT3N3b gastric cancer.


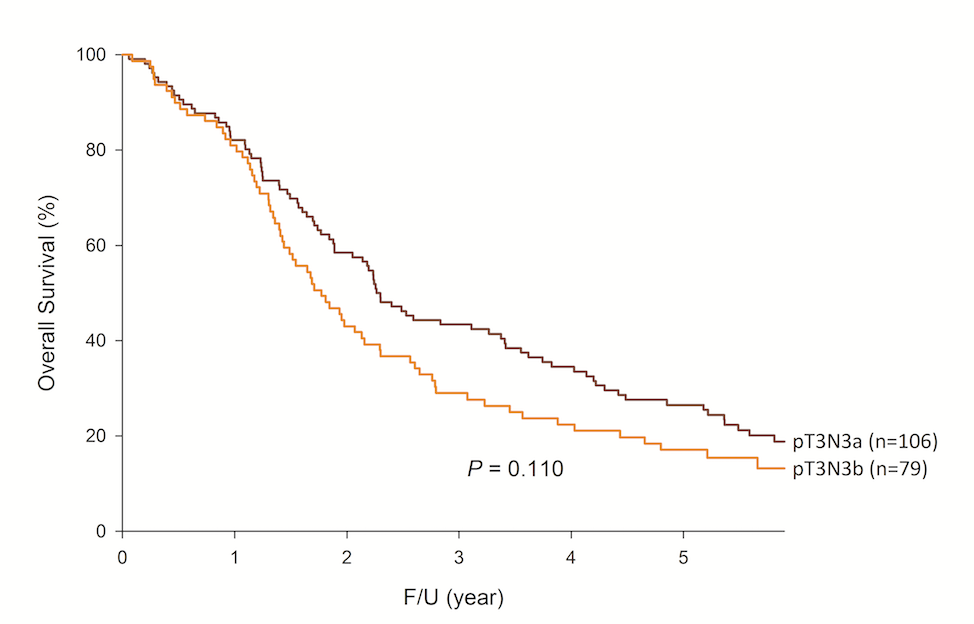

Supplement: S1 Fig — (DOCX) [file pone.0187626.s001.docx]

**S2 Fig.** The 5-year overall survival rates for pT4aN1, pT4aN2, pT4aN3a, and pT4aN3b gastric cancer.


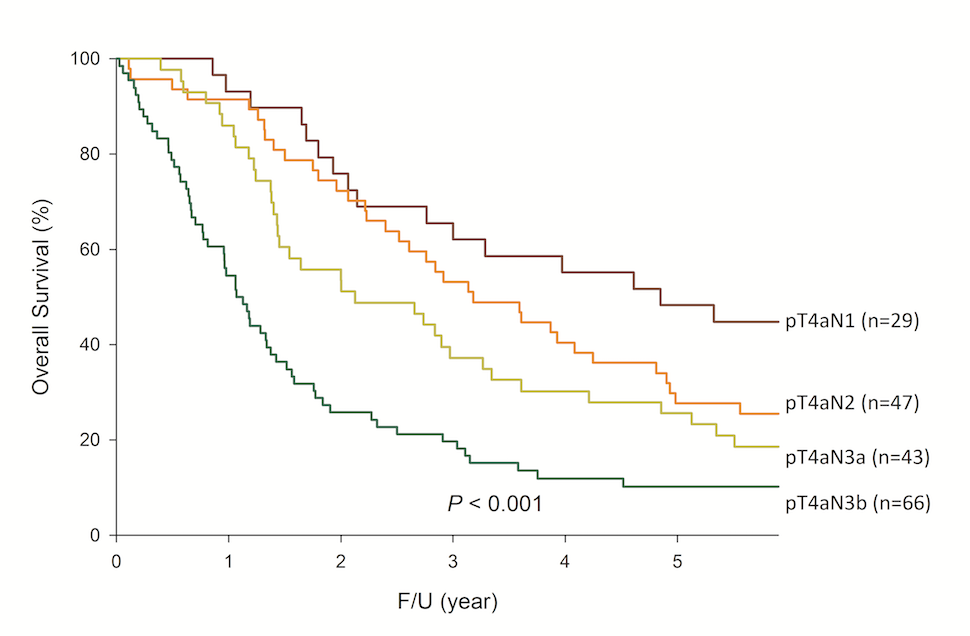

Supplement: S2 Fig — (DOCX) [file pone.0187626.s002.docx]
